# Pflegeethik und der Auftrag der Pflege – Gegenwärtige Grenzen am Beispiel der stationären Altenpflege

**DOI:** 10.1007/s00103-023-03696-2

**Published:** 2023-04-21

**Authors:** Sabine Wöhlke, Annette Riedel

**Affiliations:** 1 Gesundheitswissenschaften und Ethik, Fakultät Life Science, Hochschule für Angewandte Wissenschaften Hamburg, Campus Bergedorf, Ulmenliet 20, 21033 Hamburg, Deutschland; 2 Soziale Arbeit, Bildung und Pflege, Hochschule Esslingen, Esslingen, Deutschland

**Keywords:** Pflegeethik, Stationäre Altenpflege, Personalmangel, Deprofessionalisierung, Würdevolle Pflege, Nursing ethics, Elderly care, Staff shortage, Deprofessionalization, Dignified care

## Abstract

Zentrale ethische Prinzipien professioneller Pflege sind Würde, Fürsorge, Gerechtigkeit und Respekt. Die Rahmenbedingungen und Gegebenheiten in der Altenpflege führen aktuell dazu, dass eine professionelle Pflegeethik vielfach nicht mehr realisierbar ist. Dies führt nicht nur zu (moralischen) Belastungen bei den Pflegenden, sondern in umfassendem Maße auch zur Berufsunzufriedenheit und zum Berufsausstieg. Der Begriff „Pflexit“ wurde erstmals in der Coronapandemie laut und ist seither nicht mehr verhallt.

Um eine ethisch begründete und würdevolle wie auch an den Menschenrechten orientierte Altenpflege sicherzustellen, wie es politisch in Chartas vermittelt und zu Recht von den pflegebedürftigen Menschen erwartet wird, bedarf es der schnellen und umfassenden gesellschaftlichen und politischen Intervention. Würde und Respekt sind in diesem Zusammenhang auch ein gesellschaftlicher Auftrag. Eine professionelle, an ethischen Werten orientierte Pflege ist nur umsetzbar, wenn auch Pflegenden eben diese Haltung entgegengebracht wird. Den deutlichen Warnhinweisen auf eine „Pflegekrise“ müssen endlich Taten folgen, auch um die Berufsflucht zu stoppen. In diesem Diskussionsartikel wird zunächst die Bedeutsamkeit einer professionellen Pflegeethik dargelegt. In einem zweiten Schritt werden Rahmenbedingungen und aktuelle Probleme aufgezeigt, die einer umfassenden Umsetzung zentraler Werte in der Altenpflege entgegenstehen. Dabei wird der Schwerpunkt auf die Auswirkungen der prekären Personalsituation gelegt.

## Abstract (en)

The central guiding ethical principles of professional care are dignity, care, justice, and respect. The current framework conditions and circumstances in the care of the elderly mean that professional care ethics are no longer feasible in many cases. This leads not only to enormous (moral) burdens among the nursing staff, but also to a comprehensive degree to professional dissatisfaction and to leaving the profession. The term “Pflexit” (based on the German word “Pflege” = care) was first raised during the corona pandemic and has not faded. In order to ensure ethically justified and dignified care for the elderly that is also oriented towards human rights, as is conveyed politically in charters and rightly expected by people in need of care, rapid and comprehensive social and political intervention is required.

In this context, dignity and respect are also a social mandate. Dignified professional care based on ethical values can only be implemented if nurses are shown this same respect. The clear warnings of a “nursing climate crisis” must finally be followed by action to stop the exodus from the profession. In this discussion paper, the importance of a professional care ethic is first explained. In a second step, the framework and current problems that oppose a comprehensive implementation of core values in nursing care for the elderly are highlighted. The focus here is on the effects of the precarious personnel situation.

## Einleitung

In Zeiten großer Erschöpfung durch eine Pandemie, eines Krieges in Europa und zunehmender Folgen des Klimawandels ist die Bedeutung einer Ethik der Pflegeberufe relevanter denn je. Während der COVID-19-Pandemie sind zahlreiche vernachlässigte Themen der Sozial- und Gesundheitspolitik der letzten 20 Jahre zutage getreten, so auch die Situation der Pflege in Deutschland. Die Bedeutung einer Ethik der Fürsorglichkeit und Menschenwürde in der stationären Altenpflege wurde eindrücklich vor Augen geführt [1–5].

Pflege als ein Bereich des breiten Ensembles der Gesundheitswissenschaften legt das Interesse auf individuelle Gesundheits- und Krankheitsverläufe sowie die Ableitung von setting- und akteursspezifischen Versorgungsstrukturen, die den persönlichen Bedürfnissen und aktuellen Bedarfen Rechnung tragen. Die jeweils individuellen Pflegemaßnahmen werden hierbei mit dem pflegebedürftigen Menschen situations- und lebensweltbezogen abgewogen, an seinen Werten ausgerichtet und in einem respektvollen Aushandlungsprozess festgelegt. In diesem Zusammenhang umfasst die professionelle Pflege stets ein Beziehungs- und Interaktionsgeschehen als ein grundlegendes Merkmal professioneller Pflege [6]. Es geht folglich im Kern um Pflege- und Sorgebeziehungen [7, 8], aber auch um Verantwortungsbeziehungen, die seitens der Pflegefachpersonen in den Mittelpunkt des Entscheidens und Handelns rücken.

Care-Ethik ist ein Ansatz der Pflegeethik, der Sorge als Anteilnahme stärkt. Darüber hinaus geht es um ein Dem-anderen-zugewandt-Sein, aber auch um Mitgefühl sowie um professionelle Verantwortung in der Versorgung von Menschen.

„Care“ enthält in diesem Ansatz auf der Seite der Pflegenden auch das Element der Selbstsorge, die essentiell ist, wenn sich die Bedingungen des Sorgens verbessern und die Strukturen, die die Sorgetätigkeiten prägen, positiv verändern sollen. Care beinhaltet diesem Ansatz entsprechend auch eine achtsame Reflexion potenzieller Machtstrukturen in der Beziehung zwischen Sorgenden und Sorgeempfangenden [9].

Care als gelebte ethische Praxis fordert demgemäß die Ausgewogenheit fürsorglicher und achtsamer Tätigkeiten im Rahmen der Pflegebeziehung, die Anerkennung von Abhängigkeiten und ein hohes Maß an Verantwortlichkeit [8]. Erst diese Kombination ermöglicht eine professionelle Pflege und zugewandte Sorgebeziehung, die sich am Gegenüber, das heißt an den persönlichen Werten und höchst individuellen Bedürfnisse wie auch situativen Bedarfe des meist vulnerablen Gegenübers, ausrichtet.

Grundlegend für das ethische Handeln der Pflegenden ist der Ethikkodex für Pflegefachpersonen des International Council of Nurses (ICN; [10]). Dieser Kodex ist Ausdruck von Wertsetzungen einer Profession und stellt nachfolgend einen wichtigen Bezugspunkt dar. Der professionelle Anspruch, diesen umfassenden Anforderungen an professionelles und ethisch begründetes Handeln gerecht zu werden, führt nicht nur in Zeiten der Ungewissheit und Krisen zu Unsicherheit und Kontroversen [11]. Aufgrund dessen bildet der Ethikkodex in den nachfolgenden Ausführungen einen zentralen Bezugspunkt.

In diesem Diskussionsartikel wird zunächst die Bedeutsamkeit einer professionellen Pflegeethik dargelegt. In einem zweiten Schritt werden Rahmenbedingungen und aktuelle Probleme aufgezeigt, die einer umfassenden Umsetzung zentraler Werte in der Altenpflege entgegenstehen. Dabei wird der Schwerpunkt auf die Auswirkungen der prekären Personalsituation gelegt. Hierbei steht die Frage im Mittelpunkt, ob der Anspruch an eine ethisch reflektierte Pflegepraxis unter den aktuellen Arbeitsbedingungen in der stationären Langzeitpflege noch umsetzbar bzw. lebbar ist. Der Beitrag soll angesichts der aktuellen Gegebenheiten zur Diskussion, aber auch zum Nach‑, Neu- und Umdenken bezüglich der Fürsorge sowohl für die pflegebedürftigen Menschen als auch für die Pflegenden in den Einrichtungen der Altenpflege anregen.

## Pflegeethik und ihre Bedeutsamkeit in der Praxis

Der Kern einer ethischen Identität in der Pflege ist im ICN-Ethikkodex für Pflegefachpersonen niedergelegt [10]. Er stellt für professionell Pflegende eine allgemeinverbindliche Beschreibung der ethischen Grundhaltung und der daraus resultierenden Verpflichtungen dar. So wird das Ziel des Kodex wie folgt formuliert: „Der ICN-Ethikkodex für Pflegefachpersonen ist eine Erklärung der ethischen Werte, Aufgaben und professionellen Verantwortlichkeiten von Pflegefachpersonen und Studierenden/Lernenden in der Pflege. Er bietet eine Definition und Leitlinien für die ethische Pflegepraxis in verschiedenen Pflegerollen“ [10].

Der Ethikkodex – wie auch andere Berufskodizes – entlässt Pflegende „nicht aus ihrer Verantwortung für das Führen einer zugehörigen ethischen Diskussion“, vielmehr ist er eine „Richtschnur“ für die Verwirklichung ethischer Verantwortung und ethischen Verhaltens [12]. So wird in dem Ethikkodex [10] formuliert: „Pflegefachpersonen sind persönlich zuständig und verantwortlich für eine ethische Pflegepraxis“, sowie: „Pflegefachpersonen tragen zu einer positiven und ethischen Arbeitsumgebung bei und setzen sich gegen unethische Praktiken und Umfelder ein.“ Diese beiden Zitate untermauern den genuinen Auftrag der Pflegefachpersonen in Bezug auf eine praktizierte Pflegeethik und eine gelebte Ethik in der Pflegepraxis.

Der Kodex verweist in seiner Präambel auf eine deutliche Menschenrechtsorientierung: „Der Pflege inhärent ist die Achtung der Menschenrechte“ [10]. Das erste Element verdeutlicht die Verantwortung der Pflegefachpersonen gegenüber den pflegebedürftigen Menschen: „Die primäre berufliche Verantwortung der Pflegefachpersonen besteht gegenüber Menschen, die jetzt oder in Zukunft Pflege benötigen“ [10]. Zugleich fordert der Ethikkodex Pflegefachpersonen dazu auf, Verantwortung für die eigene Kompetenzentwicklung zu übernehmen und „ihre eigene Würde, ihr Wohlbefinden und ihre Gesundheit“ wertzuschätzen [10]. Neu ist, dass der Ethikkodex auch die Verantwortlichkeiten gegenüber der globalen (Umwelt‑)Gesundheit und dem Klimawandel aufgreift. Hervorzuheben ist, dass in allen Elementen des Kodex eine geteilte Verantwortung der Pflegefachpersonen und Führungspersonen, der Lehrenden und Forschenden und der nationalen Pflegeberufsverbände abgebildet ist.

Der Ethikkodex für Pflegefachpersonen kann als zentraler Wertekompass bezeichnet werden, da er die Werteorientierung, Haltung und ethische Verantwortung für die professionelle Berufsausübung konturiert. Folglich ist die Pflegeethik gerahmt, die konkrete Umsetzung in der Pflegepraxis indes noch offen. Die Bedeutsamkeit praktizierter Ethik in der (Alten‑)Pflege wird im Folgenden näher ausgeführt.

Angesichts der jeweiligen situativen Vulnerabilität des (alten, kranken, pflege- und hilfebedürften) Menschen sind Pflegefachpersonen verpflichtet, die Menschenwürde und Menschenrechte als zentrale normative Orientierungsdirektiven zu realisieren und den Menschenwürde- und Menschenrechtsbezug im Rahmen des professionellen Pflegehandelns sicherzustellen bzw. einzufordern [10, 13–15]. Die Verletzlichkeit von Menschen mit Demenz [13, 16, 17] – als eine der zentralen Zielgruppen professioneller Altenpflege – fordert ein höchst sensibles und achtsames Beziehungs- und Interaktionsgeschehen [6] professioneller Sorgebeziehungen [18, 19], wie dies auch der oben genannten Care-Ethik zugrunde liegt [6, 8]. Die im ICN-Ethikkodex geforderte Menschenrechtsorientierung, aber auch Verlässlichkeit, Empathie und Respekt [10] sind in der Pflege und Begleitung von Menschen mit Demenz grundlegend. Zentral für diese im ICN-Ethikkodex formulierte Haltung ist die Nichtobjektivierbarkeit des Menschen als Person als Voraussetzung einer wechselseitigen Anerkennung, die allerdings in der praktischen Umsetzung einer professionellen Pflege von Menschen mit Demenz oftmals faktisch nicht gegeben ist [20]. Die wechselseitige Anerkennung der Menschen als Person – auch und im Besonderen bei Menschen mit Demenz – liegt in der Anerkennung der Unantastbarkeit der menschlichen Würde [21, 22]. Professionelle Pflege heißt im Kontext der Altenpflege insbesondere auch, verantwortungsvoll eine Balance zwischen der Fürsorge und der (relationalen) Autonomie [15, 18] zu realisieren und eine von Selbstbestimmung geprägte Sorge [19] zu eröffnen. Speziell in der professionellen (Alten‑)Pflege und angesichts der Vielzahl in die Pflege involvierter Personen ist ein an dem pflegebedürftigen, vielfach höchst vulnerablen Menschen, an seiner Lebensqualität und an seinen Wünschen und Bedürfnissen ausgerichtetes Entscheiden und Handeln evident. In diesem Kontext kann die eingangs umrissene Care-Ethik als fürsorgliche und achtsame Praxis, „als Anwältin derjenigen verstanden werden, die sich in einer schwächeren Position befinden“ [8].

Vor dem Hintergrund der vielfältigen Situationen mit moralischem Gehalt ist es die Aufgabe der Pflegefachpersonen, ethisch bedeutsame und reflexionswürdige Situationen ethisch sensibel, konsequent und differenziert zu antizipieren, wahrzunehmen, systematisch zu analysieren und (retrospektiv) zu reflektieren sowie verantwortungsvoll ethisch gut begründete Entscheidungen zu treffen [23–26]. So fordert die professionelle (Alten‑)Pflege eine dezidierte pflegefachliche Expertise und eine ethisch professionelle Haltung. Ethische Reflexion ist hierbei kein Selbstzweck [27, 28], vielmehr kann nur so eine Haltungsveränderung im Verlauf oder im Nachgang einer systematischen (ethischen) Reflexion dazu führen, das (eigene) zukünftige Handeln situativ zu verändern oder gar neu auszurichten [23]. Aufgrund zunehmender ethischer Kontroversen in der (Alten‑)Pflege – auch angesichts der Rahmenbedingungen, der Personalsituation, -fluktuation, der Gratifikationskrise und stetigen Deprofessionalisierungsbestrebungen [29] – ist die Bedeutsamkeit ethischer Kompetenzen von Pflegefachpersonen evident.

Pflegeethik wäre allerdings zu kurzgefasst, würden wir diesen Begriff nicht zugleich in die organisatorischen, institutionellen, strukturellen und politischen Rahmenbedingungen dieser Form sozialer Interaktion einbetten [30]. Es geht also nicht nur um eine spezifische Haltung, sondern auch darum, wie die Haltung unter den aktuellen Bedingungen in einer Organisation der stationären und ambulanten Langzeitpflege möglich ist und umgesetzt werden kann. Der Begriff des „ethischen Klimas“ („ethical climate“; [30–33]) kann hier einen Bezugs- und Orientierungspunkt für die konkrete Ausgestaltung innerhalb der Organisation darstellen [30].

Die folgenden exemplarischen Ausführungen richten den Fokus darauf, inwiefern Pflegeethik in der stationären Altenpflege unter den aktuellen Rahmenbedingungen und Gegebenheiten realisiert und in einem ethischen Klima verwirklicht werden kann. Es wird expliziert, welche wesentliche Rolle die Pflegeethik in Anbetracht des Fachkräftemangels [34–36], der verschiedenen (und vielfach krankmachenden) Formen der Belastung [37–39], aber auch hinsichtlich gravierender Mängel der Arbeitsqualität [38, 39], einer verminderten Arbeitszufriedenheit [37] sowie der Gratifikationskrise [39], die aktuell in den Pflegeberufen zu konstatieren sind und vielfach die Versorgungsqualität reduzieren, spielt.

## Problemaufriss und Konsequenzen für die Realisierung der Pflegeethik

Im Pflegealltag nehmen Themen wie Sterben, Tod und Trauer einen großen und zentralen Bereich ein. Im Rahmen der Care-Ethik sollten Pflegende allerdings nicht nur als Opfer ungünstiger Bedingungen betrachtet werden, sondern auch als Handlungssubjekte, die aus einer reflektierenden Distanz heraus die Situation beurteilen können. So sollte es ihnen auch möglich sein, die Notwendigkeit von Veränderungen zu erfassen und geeignete Schritte in die Wege zu leiten [21]. Das „ethische Klima“ in der stationären Altenpflege ist allerdings aktuell weit davon entfernt, Pflegende in ihrer Autonomie und somit auch in ihrer pflegerischen Verantwortung zu stärken [40].

Autonomie bedeutet zunächst einmal, dass eine Pflegefachperson in selbstverantwortlicher Weise ihr professionelles Handeln lenkt. Es bedeutet jedoch nicht, dass sie dies unabhängig von anderen tun kann. Pflegende stehen in ihren Handlungen immer in Beziehungen mit wechselseitiger Abhängigkeit. Daraus folgt eine wechselseitige Anerkennung, der die moralische Forderung nach Achtung innewohnt [22]. Grundsätzlich ist Autonomie für Pflegende und zu Pflegende ein zentrales Prinzip, das allerdings durch institutionalisierte Abläufe, Routinen und überbordende Bürokratie gefährdet ist. Eine als gering wahrgenommene Autonomie ist vielfach das Resultat einer systematischen Deprofessionalisierung der letzten Jahrzehnte. Eine gelingende Sorgearbeit kann allerdings neben professionellen Standards nur durch ein hohes Maß an individueller Beziehungs- und Emotionsarbeit getragen werden. Diese beinhaltet eine körperbezogene, sehr persönliche Interaktion, die einer besonderen ethischen Sensibilität bedarf, da sie oftmals an den „Grenzen der Tabuverletzung, der Überschreitung individueller Grenzziehungen, an den Grenzen des Zumutbaren, der Scham, des Ekels und der eigenen Betroffenheit stattfindet“ [41]. Pflegende sehen in dieser professionell praktizierten ethischen Sensibilität den zu pflegenden Menschen als Person und wahren seine Würde. Abwehrreaktionen werden situations- und individuumbezogen gedeutet. Gelingen kann diese anspruchsvolle Pflege nur mit einem professionellen Selbstverständnis dessen, was der zentrale Kern einer würdevollen und menschenrechtsorientierten Pflege ist und gleichzeitig den Würdebergriff ins Zentrum des pflegerischen Handelns stellt.

Im Folgenden werden wir einige aus unserer Sicht zentrale Probleme benennen, die entscheidenden Einfluss darauf haben, dass Pflegende, die aktuell in der stationären Altenpflege arbeiten, ihr eigenes pflegerisches und ethisches Selbstverständnis nicht mehr benennen und auch nicht mehr realisieren können. Dieses professionelle Selbstverständnis ist allerdings, wie bereits aufgezeigt, die Grundvoraussetzung für ein pflegeethische Haltung, an der sich das tägliche professionelle Handeln im Umgang mit zu Pflegenden und Kolleg*innen ausrichtet. Die aktuellen Gegebenheiten in der stationären Altenpflege stehen diesen Anforderungen an das professionelle und ethisch begründete Pflegehandeln, im Sinne der Würde der älteren Menschen wie auch im Sinne der Berufszufriedenheit der Pflegenden, diametral entgegen [15]. Hier spielt neben dem Personalmangel, den Deprofessionalisierungsbestrebungen, -realitäten und der Gratifikationskrise vielfach auch die enorme Belastung der Pflegenden selbst eine Rolle [34, 37, 39, 42].

Dass den professionellen Ansprüchen an eine menschenrechtskonforme Versorgung der älteren pflegebedürftigen Menschen nicht Rechnung getragen werden kann, zeigt sich in unterschiedlichen ethischen Dilemmata und Konfliktsituationen im pflegerischen Alltag. Wenn diese allerdings nicht erkannt oder bearbeitet werden, wenn im Team kein Klima der ethischen Reflexion und Bearbeitung herrscht, wirkt sich dies in erheblichem Maße auf die Pflege- und Versorgungsqualität, auf die Pflegenden und deren Gesundheit wie auch auf den Berufsverbleib aus [34, 37, 39, 42]. Anhand der Beschreibung zweier aktueller Problembereiche (Brennpunkte) wollen wir verdeutlichen, wie Pflegende in Konflikt mit ihren professionellen pflegeethischen Ansprüchen und Anforderungen geraten, um damit die aktuell herausforderungsvolle Situation in der Altenpflege und den dringenden Handlungsbedarf aufzuzeigen.

### Brennpunkt 1: Die prekäre Personalsituation

Die zu geringe Bezahlung, schlechte Arbeitsbedingungen, wenig Mitbestimmungs- und Gestaltungsraum, die Gratifikationskrise, all diese Faktoren sind seit Jahrzehnten bekannt und haben zu einer kontinuierlichen Verschlechterung der Personalsituation in der stationären Altenpflege geführt [35, 39, 43]. Pflegende berichten beispielsweise, für mehr als 50 zu pflegende Menschen allein mit 2 Pflegehelfer*innen die Verantwortung tragen zu müssen. Eine solche Situation scheint immer häufiger keine Ausnahme, sondern Alltagsrealität zu sein [34].

Angesichts der prekären und äußerst instabilen Personalsituation können professionell Pflegende ihrer Fürsorgepflicht gegenüber den zu Pflegenden nicht mehr nachkommen. Sie müssen zum Beispiel die (ungerechte) Entscheidung treffen, wem sie ihre begrenzte Aufmerksamkeit schenken und wem nicht. Sie müssen entscheiden, welchen Hilferufen sie nicht nachkommen, welche zu Pflegenden sie allein in ihrem Zimmer lassen (und damit isolieren). Sie müssen eine Wahl treffen, wem sie beim Anreichen von Flüssigkeit behilflich sind, wen sie beim Essen unterstützen, wer noch aus dem Bett in einen Rollstuhl mobilisiert wird und für wen weder Zeit noch Kraft vorhanden ist, sich umfassend zuzuwenden. Dabei wird mehr als deutlich, dass Pflegende nur noch wenig berufliche Autonomie wahrnehmen. Dies steht im Widerspruch zum ICN-Ethikkodex, in dem es heißt: „Pflegefachpersonen zeigen professionelle ethische Werte wie Respekt, Gerechtigkeit, Empathie, Verlässlichkeit, Fürsorge, Mitgefühl, Vertrauenswürdigkeit und Integrität“ [10]. Diese menschlichen Tugenden sind es, über die den zu Pflegenden in ihrer jeweiligen Situation Würde vermittelt wird.

Die täglichen Entscheidungen gegen notwendige Pflegemaßnahmen und in der Folge auch gegen eine würdevolle Pflege verursachen moralischen Stress, das heißt, Pflegende müssen Pflegehandlungen gegen ihre eigenen moralischen Überzeugungen durchführen oder unterlassen [23, 44]. Pflegende können dem moralischen Stress vielfach weder verbal noch auf irgendeine andere Weise begegnen, wenn ihnen die hierfür notwendige ethische Reflexions- und Handlungskompetenz und der entsprechende organisationsethische Rahmen fehlt.

Würdevolle Pflege kann nur gelingen, wenn man das Wesen des anderen anerkennt und ihm als Menschen begegnet. Würdevoll zu sein und zu handeln beinhaltet, das professionelle Handeln an den Grundbedürfnissen des Menschen auszurichten, aber auch die eigenen Gefühle „wahr-zu-nehmen“ und zu reflektieren. Wenn professionelle Pflege, wie aufgezeigt, nicht mehr realisierbar ist und Pflegefachpersonen ihren Kernkompetenzen im Pflegealltag nicht mehr nachkommen können, werden – um dem moralischen Stress zu entfliehen – eigene „Überlebensstrategien“ genutzt, wie etwa die Vermeidung bestimmter Situationen bis hin zu Flucht oder Abwehrhaltungen. Dabei können Pflegende in einem zunehmend menschlich verrohenden Pflegealltag, in dem es zu Beschimpfungen und Mobbing im Team kommen kann, selbst verletzlich sein, so dass sie sich schützen müssen [45]. Studien zeigen einen Zusammenhang zwischen moralischem Stress und negativen Emotionen [44]. Pflegende werden dabei in ihrer Selbstwahrnehmung durch Selbstzweifel, Schuldgefühle, Scham, Ekel, Verlegenheit, Wut sowie vermindertes Selbstvertrauen stark beeinflusst. Dies wiederum hat Einfluss auf die Berufszufriedenheit und den Berufsverbleib. Denn: Um sich selbst zu schützen wird es zudem vermieden, das eigene Pflegehandeln zu reflektieren. Dies alles hat zur Folge, dass dem zu pflegenden Menschen meist nicht mehr mit angemessener Würde begegnet werden kann. Um sich selbst zu schützen, bleibt häufig nur noch der Weg raus aus dem Beruf [26].

### Brennpunkt 2: Die zunehmende Deprofessionalisierung (zu wenig ausgebildetes Personal)

Von den 1,7 Mio. Beschäftigten im Pflegebereich sind 63 % Fachkräfte, 8 % Spezialist*innen und 29 % Helfer*innen [46]. In den stationären Pflegeeinrichtungen sehen diese Zahlen allerdings deutlich anders aus. Hier sind es nur noch 49 % Fachkräfte und bereits 47 % Helfer*innen. Nur 4 % der Pflegenden verfügen über eine Zusatz- bzw. Fachausbildung. Die Pflegehelfer*innen spielen laut Statistischem Bundesamt für die Beschäftigungsentwicklung in stationären Pflegeeinrichtungen eine wichtige Rolle. Ihre Zahl ist im Vergleich zum Jahr 2016 um 40.000 gestiegen [46].

Dieses zunehmende Missverhältnis zwischen Pflegefachpersonen und Hilfskräften führt dazu, dass die Zuständigkeiten und Verantwortlichkeiten zusehends an Trennschärfe zwischen beiden Qualifikationsgruppen verlieren und dass Pflegequalität und Berufszufriedenheit sinken. In einem aktuellen Beitrag zur Situation der professionellen Ausbildung in der Pflege wird ein intransparentes Qualifizierungslevel der Assistenzpersonen kritisiert. So existieren aktuell bundesweit 27 unterschiedliche ein- bis zweijährige Pflegehelferausbildungen, die in 8 unterschiedlichen Berufsbezeichnungen münden [38]. Kompetenz- und Handlungsprofile bleiben hierbei undurchsichtig und stellen ein enormes Gefährdungspotenzial für die zu Pflegenden dar.

Bei einer fehlenden Qualifikationstiefe kann eine pflegeethische Grundhaltung nicht mehr gewährleistet werden. Unzureichend qualifizierte Pflegehelfer*innen können die pflegerischen Bedarfe häufig nicht erkennen und Fehlverhalten sowie Fehler nicht wahrnehmen und reflektieren, wodurch die zu Pflegenden unzureichend versorgt und aus ethischer Sicht in ihrer menschlichen Würde verletzt werden können.

Für eine professionelle und menschenrechtsorientierte Pflege und die damit verbundene Pflegebeziehung sind Vertrauen und eine vertrauensvolle Atmosphäre notwendig [47, 48]. Die aktuelle Personalsituation und -konstellation lässt es allerdings kaum noch zu, dass eine vertrauensvolle Atmosphäre entstehen kann, und stellt aus unserer Sicht ein wesentliches Hemmnis für die Umsetzung einer gelingenden pflegeethischen Haltung dar.

Zugleich bleibt in der aktuellen Personalsituation für die Pflegefachpersonen kaum noch Zeit für eine umfassend wertschätzende und würdevolle Pflege. Es sind neue Aufgaben hinzugekommen, wie zum Beispiel die Anleitung von Hilfskräften, die Kontrolle delegierter Tätigkeiten, die Qualitätssicherung einer Pflege unter restriktiven und unangemessenen Rahmenbedingungen. Das heißt, die Folge des jahrzehntelangen Versuchs, die dünne Personaldecke mit (Pflege‑)Helfer*innen/Alltagsbegleiter*innen etc. zu füllen, führt dazu, dass die ohnehin vielerorts sich in der Minderzahl befindenden Pflegefachpersonen vornehmlich damit beschäftigt sind, zu delegieren und zu kontrollieren, um damit dem Arbeitsschutz gerecht zu werden und eine wie auch immer sich rechtfertigende Qualitätssicherung durchzuführen.

Aus ethischer Sicht wird hier ein umfassendes strukturelles Defizit sichtbar. Der gesamte Pflegesektor wird durch ein Personalmanagement strukturiert und organisiert [22], das vielfach der Logik der Marktwirtschaft folgt [49]. Dem gesamten Bereich der Planung, Strukturierung und Umsetzung von Personalfragen im Pflegebereich liegen ökonomische Kriterien zugrunde. Das immaterielle Gut „Care- bzw. Sorgearbeit“ als emotionsbezogene und professionelle Pflegehandlung ist allerdings nicht Teil dieser Logik bzw. der Ressourcenallokation. Es wird deutlich, dass im Bereich der Personalplanung (ein spezieller Bereich des Pflegemanagements und der Pflegewissenschaft selbst) Lösungen im Sinne einer Basalversorgung geschaffen worden sind, die die Zahl ethischer Konfliktsituationen für die Pflegefachpersonen weiter erhöht.

Beide Brennpunktbeschreibungen machen deutlich, dass Pflegeethik und die ethisch verantwortungsvolle Reflexion der Pflegehandlungen in der Alltagsrealität der stationären Altenpflege vielfach keinen „Platz“ mehr haben und in der Emotions- und Sorgearbeit nicht (mehr) als Kern präsent sind.

## Ausblick: Die Perspektive auf die Pflege, die pflegebedürftigen Menschen und die Pflegenden richten und ein Umdenken in Gesellschaft und Politik fördern

Die Gesellschaft und mit ihr die Bundespolitik stehen in der Verantwortung, sich dem Thema Pflege grundständig zuzuwenden, um die aktuelle Situation in ihrer Vielschichtigkeit zu erfassen und notwendige Reformen einzuleiten. Es ist zu klären, welchen Wert wir der Pflege und Versorgung von alten und kranken Menschen in unserem Land beimessen [50]. Die Gesellschaft trägt die Verantwortung dafür, dass sich die Bedingungen für die Pflege körperlich oder psychisch beeinträchtigter älterer Menschen hierzulande zunehmend verschlechtern. Und sie ist es, die die nötigen Bedingungen schaffen kann, um dem Würdebegriff im Pflegesektor wieder mehr gerecht zu werden.

Die Pflege älterer Menschen mit dem Anspruch an eine pflegerische Grundhaltung, für die das leitende Prinzip der menschlichen Würde evident ist, erfordert spezifische Kompetenzen. Insbesondere bei älteren Menschen mit demenziellen Erkrankungen wird der Würdebezug aufgrund der eingeschränkten kognitiven Fähigkeiten und der damit verbundenen Pflegebedürftigkeit zentral. Der Würdebegriff wird in der Medizinethik oft mit dem „respect for autonomy“ gleichgesetzt [51]. Pflegeethik bezieht durch den Ansatz der relationalen Autonomie die sorgenden Personen mit ein, sie sind Teil des Würdeverständnisses. Die Erweiterung auf die Sorgenden bezieht in pflegebezogenen Beziehungen auch die Pflegenden selbst mit ein. Diese fachlichen und ethischen Forderungen und der damit verbundene professionelle Anspruch benötigen entsprechende personelle Rahmenbedingungen und organisationale wie auch organisationsethische Strukturen.

Parallel zu einer dezidierten konzeptionellen und kompetenzbezogenen Verbesserung ist die gezielte Forschung im Bereich der stationären Altenpflege dringend geboten. Um das Leben, den Alltag, das Miteinander mit den zu pflegenden Menschen und den Kolleg*innen besser zu verstehen, bedarf es praxisnaher qualitativ sozialempirischer Langzeitstudien, die die aktuelle Lebenswelt der Menschen in der stationären Altenpflege sowie des Pflegepersonals sichtbar und erfahrbar machen. Nur so wird es möglich sein, realistische Wege zu finden, die Pflegenden wieder in die Lage versetzen, eine ethische Grundhaltung im Rahmen der professionellen Pflege einzunehmen. Pflegende und zu pflegende Menschen sollten durch Forschungsansätze der partizipativen Forschung aktiv in die Forschung einbezogen werden. Dabei sollen sie nicht nur (passiv) beforscht werden, sondern aktiv mitwirken und mitbestimmen. Im Sinne von Partizipation ist die Teilhabe nicht an die Kompetenz eines Einzelnen gebunden, zentral ist ein soziales Zusammen-Gestalten. Mithilfe dieser Ansätze, die eine wertschätzende Grundhaltung voraussetzen, lassen sich die zentralen Werte der Sorgearbeit herausarbeiten und für die zukünftige Pflege im Sinne der Qualitätsverbesserung aufbereiten.
